# Supplementary material for: First Complete Mitochondrial Genome of Melyridae(Coleoptera, Cleroidea): Genome Description and Phylogenetic Implications
Source: Insects. 2021 Jan 20;12(2):87. doi: 10.3390/insects12020087 (PMC7909412; doi:10.3390/insects12020087)
Supplement: Supplementary file 1 [file insects-12-00087-s001.pdf]

insects MDPI

Table S1. The optimal partition schemes and the best-fit replacement models for the Bayesian Inference (BI)

Partitions	Models	Genes
P1	GTR+F+I+G	atp6_pos1,cox2_pos1,cox3_pos1,cytb_pos1,nad3_pos1
P2	GTR+F+I+G	atp6_pos2,cox1_pos2,cox2_pos2,cox3_pos2,cytb_pos2
P3	HKY+F+I+G	atp6_pos3
P4	GTR+F+I+G	atp8_pos1,nad2_pos1,nad6_pos1
P5	GTR+F+G	atp8_pos2,nad2_pos2,nad3_pos2,nad6_pos2
P6	HKY+F+G	atp8_pos3,cox1_pos3,cox2_pos3,cox3_pos3,cytb_pos3
P7	GTR+F+G	cox1_pos1
P8	GTR+F+I+G	nad1_pos1, nad4_pos1, nad14l_pos1, nad5_pos1
P9	GTR+F+I+G	nad1_pos2, nad4_pos2, nad14l_pos2, nad5_pos2
P10	HKY+F+G	nad1_pos3
P11	HKY+F+G	nad2_pos3
P12	HKY+F+G	nad3_pos3
P13	HKY+F+G	nad4_pos3
P14	HKY+F+G	nad4l_pos3
P15	HKY+F+G	nad5_pos3
P16	HKY+F+G	nad6_pos3

Table S2. The optimal partition schemes and the best-fit replacement models for the Maximum Likelihood (ML)

Partitions	Models	Genes
P1	GTR+F+I+G	atp6,cox1,cox2,cox3
P2	HKY+F+G	atp8
P3	GTR+F+I+G	Cytb,nad3
P4	TIM+F+I+G	nad1
P5	TVM+F+I+G	nad2
P6	GTR+F+I+G	nad4L
P7	GTR+F+I+G	nad4
P8	TIM+F+I+G	nad6

Table S3. Summary of the characteristics of the mitogenome of *Cordylepherus* sp.

Feature	Strand	Location	Size (bp)	Start Codon	Stop Codon	Anticodon	Intergenic Nucleotides
trnI	J	1—65	65			AAT	0
trnQ	N	65—132	68			GTA	-1
trnM	J	133—201	69			AAA	
nad2	J	202—1180	979	ATA	T		
trnW	J	1181—1245	65			AAG	
trnC	N	1255—1317	63			GAT	9
trnY	N	1320—1383	64			ATC	2

cox1	J	1418—2915	1498	ATT	T		34
trnL2	J	2916—2980	65			TCT	
cox2	J	2981—3661	681	ATA	TAA		
trnK	J	3663—3733	71			CAT	1
trnD	J	3733—3797	65			AAA	-1
atp8	J	3798—3956	159	ATC	TAA		
atp6	J	3956—4615	660	ATA	TAA		-1
cox3	J	4618—5400	783	ATA	TAA		2
trnG	J	5404—5470	67			ATT	3
nad3	J	5475—5820	346	ATA	T		4
trnR	J	5821—5886	66			AAA	
trnA	J	5890—5954	65			AGG	3
trnN	J	5955—6020	66			TTA	
trnS1	J	6021—6087	67			GAA	
trnE	J	6090—6153	64			ATT	2
trnF	N	6152—6217	66			AGT	-2
nad5	N	6218—7928	1711	ATA	T		
trnH	N	7926—7989	64			AAT	-3
nad4	N	7990—9320	1331	ATA	TA		
nad4L	N	9317—9574	258	ATA	TAA		-4
trnT	J	9595—9659	65			GTT	20
trnP	J	9660—9725	66			CTG	
nad6	J	9727—10233	507	ATA	TAA		1
cytb	J	10233—11367	1135	ATG	T		-1
tRNAS2	J	11368—11433	66			AAT	
nad1	N	11451—12398	948	TTG	TAG		17
trnL2	N	12399—12463	65			AAT	
rrnL	N	12464—13748	1285				
trnV	N	13749—13815	67			TTG	
rrnS	N	13816—14630	815				
CR	J	14631—15824	>1194				

Note: the "J" indicates the majority strand and the "N" indicates the minority strand in the strand column.

Table S4. Nucleotide composition and skewness of the mitogenomes of *Cordylepherus* sp.

	**Size (bp)**	**A**	**T**	**G**	**C**	**A + T**	**G + C**	**AT-skew**	**GC-skew**
Full genome	15,824	41.90%	39.20%	7.80%	11.20%	81.1%	19.00%	0.03	-0.18
PCGs	10,996	41.50%	38.20%	8.50%	11.80%	79.70%	20.30%	0.04	-0.16
rRNA genes	2,100	43.10%	41.40%	5.30%	10.20%	84.50%	15.50%	0.02	-0.32
tRNA genes	1,449	43.90%	37.80%	7.70%	10.60%	81.70%	18.30%	0.07	-0.16
CR	1,194	43.90%	46.10%	4.50%	5.40%	90.0%	10.0%	-0.02	-0.09

Table S5. Codon number and RSCU in the mitogenomes of Melyridae species (*Cordylepherus* sp. / *Clanoptilus assimilis* / Malachiinae sp. / Dasytinae sp.)

Codon	Count	RSCU	Codon	Count	RSCU	Codon	Count	RSCU
UUU(F)	343/331/ 327/333	1.88/1.8/ 1.82/1.86	UCU(S)	98/94/ 114/109	2.5/2.36/ 2.83/2.67	UAU(Y)	160/149/ 159/ 160	1.77/1.74 1.74/1.81
UUC(F)	22/36/ 33/26	0.12/0.2 0.18/0.14	UCC(S)	2/ 10/ 11/ 10	0.05/0.25/ 0.27/0.25	UAC(Y)	21/ 22/ 24/ 17	0.23/0.26/ 0.26/0.19
UUA(L)	502/471/ 469/475	5.11/4.79/ 4.8/ 4.9	UCA(S)	93/ 89/ 68/ 92	2.38/2.23 1.69/2.26	UAA(*)	6/ 11/ 9/ 8	1.71/1.83/ 1.8/ 1.45
UUG(L)	21/ 30/ 37 14	0.21/0.31/ 0.38/0.14	UCG(S)	3/ 6/ 4/3	0.08/0.15 0.1/ 0.07	UAG(*)	1/1/ 1/3	0.29/0.17/ 0.2/ 0.55
CUU(L)	36/ 35/ 42/ 46	0.37/0.36/ 0.43/0.47	CCU(P)	76/ 70/ 75/ 78	2.45/2.26 2.4/ 2.38	CAU(H)	58/ 55/ 58/ 60	1.78/1.59/ 1.71/1.69
CUC(L)	0/ 7/ 0/ 3	0/ 0.07/ 0/ 0.03	CCC(P)	7/ 11/ 12/ 12	0.23/0.35 0.38/0.37	CAC(H)	7/ 14/ 10/ 11	0.22/0.41 0.29/0.31
CUA(L)	29/ 46/ 35/ 44	0.3/ 0.47/ 0.36/0.45	CCA(P)	39/ 41/ 35/39	1.26/1.32 1.12/1.19	CAA(Q)	63/ 61/ 57/ 59	1.88/1.85/ 1.87/1.87
CUG(L)	1/ 1/ 3/0	0.01/0.01/ 0.03/0	CCG(P)	2/ 2/ 3/2	0.06/0.06 0.1/ 0.06	CAG(Q)	4/ 5/ 4/ 4	0.12/0.15 0.13/0.13
AUU(I)	405/398 393/372	1.91/1.85/ 1.84/1.84	ACU(T)	77/ 66/ 77/ 80	1.99/1.71 1.99/2.01	AAU(N)	205/207/ 188/193	1.83/1.86/ 1.72/ 1.8
AUC(I)	18/ 32/ 35 / 33	0.09/0.15/ 0.16/0.16	ACC(T)	4/ 14/ 13/ 13	0.1/0.36/ 0.34/0.33	AAC(N)	19/ 15 / 31/21	0.17/0.14 0.28/ 0.2
AUA(M)	265/263/ 254/270	1.89/1.79/ 1.8/ 1.9	ACA(T)	71/ 70/ 61/ 65	1.83/1.82 1.57/1.64	AAA(K)	98/ 99/ 111/104	1.8/ 1.78/ 1.82/1.78
AUG(M)	15/ 31/ 28/ 14	0.11/0.21/ 0.2/ 0.1	ACG(T)	3/ 4/ 4/1	0.08/0.1/ 0.1/ 0.03	AAG(K)	11/ 12/ 11/ 13	0.2/ 0.22/ 0.18/0.22
GUU(V)	71/ 69/ 80/ 81	2.01/1.85/ 2.27/2.08	GCU(A)	65/ 64/ 61/ 71	1.98/1.98 2.09/1.96	GAU(D)	48/ 42/ 58/ 53	1.63/1.38/ 1.73/1.68
GUC(V)	5/ 6/ 7/6	0.14/0.16/ 0.2/ 0.15	GCC(A)	10/ 20/ 12/ 14	0.31/0.62 0.41/0.39	GAC(D)	11/ 19/ 9/ 10	0.37/0.62/ 0.27/0.32
GUA(V)	60/ 65/ 46/ 61	1.7/ 1.74/ 1.3/ 1.56	GCA(A)	53/ 44/ 40/ 58	1.62/1.36 1.37/ 1.6	GAA(E)	68/ 66/ 60/ 64	1.81/1.71 1.69/1.75
GUG(V)	5/ 9/ 8/8	0.14/0.24/ 0.23/0.21	GCG(A)	3/ 1/ 4/2	0.09/0.03 0.14/0.06	GAG(E)	7/ 11/ 11/ 9	0.19/0.29/ 0.31/0.25
AGA(S)	91/87/ 83/ 82	2.33/2.18/ 2.06/2.01	AGU(S)	25/31/ 31/21	0.64/0.78 0.77/0.52	UGU(C)	27/26/ 32/ 33	1.8/1.73/ 1.94/1.94
AGG(S)	1/1/ 6/4	0.03/0.03/ 0.15/ 0.1	AGC(S)	0/1/ 5/5	0/ 0.03/ 0.12/0.12	UGC(C)	3/ 4/ 1/1	0.2/ 0.27 0.06/0.06
GGU(G)	50/32/ 38/ 35	1.1/ 0.7/ 0.84/ 0.7	CGU(R)	17/21/ 15/ 21	1.28/1.58 1.15/1.62	UGA(W)	84/83/ 89/ 91	1.87/1.82/ 1.84/ 1.9
GGC(G)	4/ 11/	0.09/0.24/	CGC(R)	0/2/	0/ 0.15/	UGG(W)	6/8/	0.13/0.18

	6/ 12	0.13/0.24		5/2	0.38/0.15		8/5	0.16/ 0.1
GGA(G)	112/100/	2.46/2.17/	CGA(R)	36/26/	2.72/1.96/			
	99/ 120	2.18/2.39		25/ 27	1.92/2.08			
GGG(G)	16/ 41/	0.35/0.89	CGG(R)	0/ 4/	0/ 0.3/			
	39/ 34	0.86/0.68		7/2	0.54/0.15			

Table S6. Length of PCGs in the mitogenomes of the Melyridae species

gene	Length of PCGs			
	***Cordylepherus* sp.**	***Clanoptilus assimilis***	**Malachiinae sp.**	**Dasytinae sp.**
atp6	660	666	666	681
atp8	159	159	156	156
cox1	1498	1545	1545	1545
cox2	681	681	679	682
cox3	783	786	786	786
cytb	1135	1137	1131	1140
nad1	948	948	942	948
nad2	979	1008	978	1011
nad3	346	357	357	357
nad4	1331	1332	1324	1336
nad4L	258	276	273	285
nad5	1711	1711	1705	1734
nad6	507	504	504	498

Table S7. AT content in the mitogenomes of Melyridae species

gene	A + T Content			
	***Cordylepherus* sp.**	***Clanoptilus assimilis***	**Malachiinae sp.**	**Dasytinae sp.**
Full genome	81.1	77.8	79.7	79.2
PCGs	79.3	77.7	77.9	77.6
tRNAs	81.7	81.9	82	80.7
1stcondon position	73.71	72.96	73.81	71.82
2ndcondon position	70.58	70.73	70.58	69.24
3rdcondon position	93.67	89.44	89.35	91.56
atp6	79.1	77.8	77.7	76.2
atp8	91.2	86.2	87.8	84.0
cox1	70.1	69.2	69.3	69.2
cox2	76.8	76.1	76.1	73
cox3	75.2	73.6	73.5	73.1
cytb	75.5	73.1	74.8	73.9
nad1	80.0	78.4	78.4	78.7
nad2	83.4	79.6	81.2	81.1

nad3	80.9	82.1	82.1	79.9
nad4	83.4	81.3	81.3	81.9
nad4L	82.6	82.9	81.3	81.4
nad5	83.3	81.7	81.1	81.5
nad6	85.4	86.1	86.1	86.2
